# Interpretable polyp classification via end-to-end Concept Bottleneck Models with vision-language concept alignment

**DOI:** 10.3389/frai.2026.1853584

**Published:** 2026-07-10

**Authors:** Qiunan Ji, Zihe Feng, Xinjuan Liu, Jianyu Hao

**Affiliations:** 1 Beijing Chaoyang Hospital, Capital Medical University, Beijing, China; 2 Armour College of Engineering, Department of Electrical and Computer Engineering, Illinois Institute of Technology, Chicago, IL, United States

**Keywords:** colonoscopy, Concept Bottleneck Model, explainable AI, polyp classification, sessile serrated lesion, vision-language model

## Abstract

**Background and objective:**

Artificial intelligence has improved adenoma detection during colonoscopy, but most models remain black boxes, limiting clinical trust, especially for sessile serrated lesions (SSLs). Concept Bottleneck Models (CBMs) route predictions through human-understandable concepts. We propose an end-to-end CBM for colonoscopic polyp classification with concept-level explanations aligned with clinical standards.

**Methods:**

We defined 62 concepts from NBI International Colorectal Endoscopic (NICE), Japan NBI Expert Team (JNET), and Paris classification systems covering color, morphology, vascular/pit patterns, and contextual features. BiomedCLIP generated pseudo concept labels by patch-level image-text cosine similarity with max-pooling; these were not expert concept annotations. A concept projection layer with ReLU activation was inserted between an EfficientNet-B3 backbone and a linear classifier. Training used cross-entropy plus mean squared error concept alignment (lambda = 1.0). Evaluation used 508 images (192 SSL, 170 conventional, 146 malignant) and 5-fold image-level stratified cross-validation.

**Results:**

The CBM achieved macro F1 of 95.20% (±1.89%; 95% CI 92.58–97.82%), comparable to the black-box baseline of 94.97% (±2.52%; 95% CI 91.48–98.47%). Paired testing showed no significant fold-level difference (paired *t*-test, *p* = 0.835; Wilcoxon, *p* = 1.000), supporting comparability rather than superiority. Alignment strength showed an overall upward trend within the CBM family (F1 92.90% at lambda = 0 to 94.39% at lambda = 1.0). Concept ablation suggested distributed concept use, with no single concept causing more than a 0.80% F1 drop. Per-class F1 was 95.11% for SSL, 92.87% for conventional polyps, and 97.63% for malignant lesions.

**Conclusions:**

Vision-language-aligned end-to-end CBM achieved interpretable polyp classification while maintaining black-box-level performance. Concept alignment may act as a regularization-like constraint, requiring further validation. This framework enables clinicians to inspect concept-level evidence for AI-assisted colonoscopy.

## Introduction

Colorectal cancer (CRC) is the third most common cancer worldwide and the second leading cause of cancer-related mortality (Sung et al., 2021). Early detection and removal of precancerous polyps during colonoscopy significantly reduces CRC incidence and mortality (Zauber et al., 2012). Artificial intelligence (AI) systems for computer-aided detection (CADe) have yielded substantial improvements in adenoma detection rates (ADR), with meta-analyses reporting relative increases of approximately 20% (Hassan et al., 2021, 2023). However, these systems operate as black boxes, providing predictions without explanations of their underlying decision-making processes (Barredo Arrieta et al., 2020).

The clinical need for interpretability is particularly acute for sessile serrated lesions (SSLs). SSLs are recognized precursors in the serrated neoplasia pathway, accounting for an estimated 20–30% of all CRCs (Rex et al., 2012). Despite their clinical significance, SSLs are notoriously difficult to detect due to their flat morphology, pale coloring, and indistinct borders (IJspeert et al., 2016). A recent large-scale meta-analysis of 28 randomized controlled trials encompassing 23,861 patients found that while AI significantly improved overall ADR, the improvement in SSL detection rate was not statistically significant (*p* = 0.27) (Makar et al., 2025). This finding highlights a critical gap: current AI systems fail precisely where clinical assistance is most needed, and without interpretability, clinicians cannot understand *why* these systems underperform.

Existing approaches to explainable AI (XAI) in medical imaging rely predominantly on *post-hoc* saliency methods such as Grad-CAM (Selvaraju et al., 2017), which generate heatmaps indicating *where* the model attends but not *what* features it recognizes. This limitation is particularly relevant in clinical settings where diagnostic decisions depend on specific morphological and vascular features defined by standardized classification systems such as NBI International Colorectal Endoscopic (NICE) (Hewett et al., 2012), Japan NBI Expert Team (JNET) (Sano et al., 2016), and Paris classification (The Paris Endoscopic Classification Review Group, 2003). Concept Bottleneck Models (CBMs) (Koh et al., 2020) address this gap by decomposing predictions into an intermediate layer of human-interpretable concepts, enabling clinicians to understand, validate, and intervene in the model's reasoning process. Recent advances have extended CBMs to label-free training with vision-language models (Oikarinen et al., 2023) and *post-hoc* concept extraction (Yuksekgonul et al., 2023), although applications in medical imaging remain limited (van der Velden et al., 2022).

In this work, we present the first application of end-to-end CBMs to colonoscopic polyp classification. Our framework classifies polyps into three clinically significant categories—SSL, conventional, and malignant—through 62 clinical concepts derived from NICE, JNET, and Paris classification standards. We leverage BiomedCLIP (Zhang et al., 2023), a biomedical vision-language model, to generate concept alignment targets without requiring manual concept annotations. Our contributions are 3-fold:

1. We propose the first end-to-end CBM for colonoscopic polyp classification with 62 clinically grounded concepts, providing structured explanations that map directly to established diagnostic criteria.
2. We show that concept alignment may act as a regularization-like constraint, enabling interpretable classification with black-box-level performance (F1: 95.20% vs. 94.97%) while improving over the no-alignment CBM variant.
3. We investigate the functional role of learned concepts through systematic intervention experiments, revealing distributed concept encoding consistent with multi-feature clinical reasoning.

## Methods

### Dataset

A total of 508 colonoscopy images were retrospectively collected from a single center. Images were categorized into three classes: sessile serrated lesions (SSL; *n* = 192; 1,280 × 720 pixels), conventional polyps (*n* = 170; 1,920 × 1,080 pixels), and malignant polyps (*n* = 146; 1,920 × 1,080 pixels). All images were resized to 512 × 512 pixels for model input. This study was approved by the Ethics Committee of Beijing Chao-Yang Hospital, Capital Medical University (acceptance number: 2024-2-25-1; approval number: 2024-Ke-78; approval date: 12 April 2024) and was conducted in accordance with the Declaration of Helsinki. Informed-consent procedures were reviewed as part of the approved study protocol.

### Clinical concept design

We designed 62 clinical concepts based on three established endoscopic classification systems: the NICE (NBI International Colorectal Endoscopic) classification (Hewett et al., 2012), the JNET (Japan NBI Expert Team) classification (Sano et al., 2016), and the Paris classification for gastrointestinal neoplasia (The Paris Endoscopic Classification Review Group, 2003). Concepts were organized into four categories across the three polyp classes:

- SSL concepts (*n* = 24): surface color features (e.g., pale whitish surface, mucus cap), morphological features (e.g., flat sessile morphology, indistinct borders), vascular/pit patterns (e.g., absence of typical vascular pattern, branching crypts), and location features.
- Conventional polyp concepts (*n* = 18): surface color (e.g., reddish surface, lobulated pattern), morphology (e.g., pedunculated with stalk, well-defined borders), vascular/pit patterns (e.g., tubular architecture, regular pit pattern), and location.
- Malignant polyp concepts (*n* = 20): surface color (e.g., ulcerated bleeding surface, heterogeneous color), morphology (e.g., irregular margins, central depression), vascular/pit patterns (e.g., disrupted vascular pattern, destroyed pit pattern), and invasion features.

Each concept was formulated as a descriptive text prompt (e.g., “a colonoscopy image showing pale whitish mucosal surface”) for compatibility with vision-language alignment.

### Concept alignment via BiomedCLIP

We employed BiomedCLIP (Zhang et al., 2023), a vision-language model pre-trained on 15 million biomedical image-text pairs from PubMed, to generate concept alignment targets without manual annotation. For each image, we computed concept scores through the following procedure:

1. Divide the input image into overlapping patches.
2. Compute the cosine similarity between each patch embedding and each of the 62 concept text embeddings using BiomedCLIP's shared latent space.
3. Apply max-pooling across patches for each concept, yielding a single score per concept per image.

This produced a concept score matrix **S** ∈ ℝ^508×62^ serving as soft alignment targets for training.

### End-to-end CBM architecture

**Figure 1:**
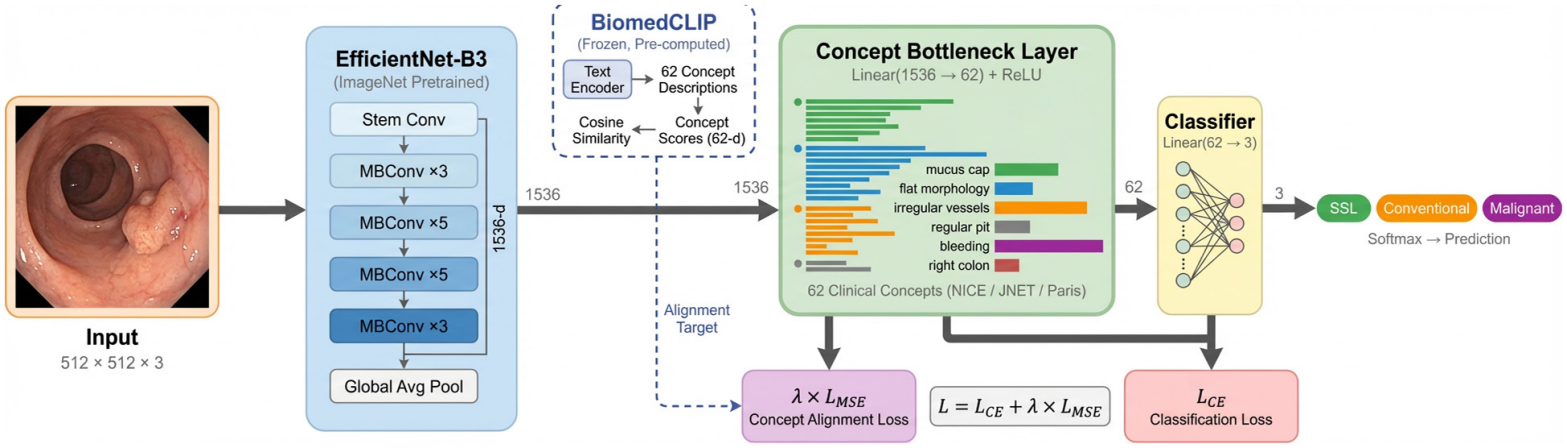
Overview of the proposed end-to-end Concept Bottleneck Model (E2E-CBM) architecture. An input colonoscopy image is processed by an EfficientNet-B3 backbone pre-trained on ImageNet. The extracted features are projected onto a 62-dimensional clinical concept space via a learned concept projection layer with ReLU activation. A linear classifier then maps the concept activations to the final polyp classification (SSL, Conventional, or Malignant). During training, the concept layer is aligned with BiomedCLIP-derived concept scores through a hybrid loss combining cross-entropy classification loss and mean squared error alignment loss weighted by λ.

Our model consists of three components (Figure 1):

1. Feature extractor: An EfficientNet-B3 backbone (Tan and Le, 2019) pre-trained on ImageNet, producing a 1,536-dimensional feature vector after global average pooling.
2. Concept projection layer: A linear layer projecting the 1,536-dimensional features to 62 concept activations, followed by ReLU activation to ensure non-negative, interpretable concept values.
3. Classifier: A linear layer mapping 62 concept activations to three class logits.

The model is trained end-to-end with a hybrid loss:


$$\begin{array}{l} ℒ\;=\;{ℒ}_{\text{CE}}\left(ŷ,y\right)+\lambda \cdot {ℒ}_{\text{MSE}}\left(c,s\right) \end{array}$$

where ℒ_CE_ is the weighted cross-entropy loss (weights inversely proportional to class frequency), ℒ_MSE_ is the mean squared error between predicted concept activations **c** and BiomedCLIP concept scores **s**, and λ controls the alignment strength.

### Training protocol

All experiments used 5-fold image-level stratified cross-validation (seed = 42) to ensure class-balanced splits. Patient-level or lesion-level grouping was not applied because patient and lesion identifiers were not available for the present analysis; we therefore explicitly treat this as a limitation of the present exploratory study. Training used the AdamW optimizer with a learning rate of 10^−4^, weight decay of 10^−4^, and cosine annealing scheduling (minimum learning rate 10^−6^). Data augmentation included random resized cropping, horizontal and vertical flips, color jitter, and random rotation. The primary configuration employed a batch size of 8, 50 training epochs, and λ = 1.0. The black-box baseline consisted of an identical EfficientNet-B3 backbone with a direct linear classifier (1,536 → 3), trained under otherwise identical conditions.

### Concept intervention protocol

To evaluate the functional role of individual concepts, we performed systematic concept intervention experiments. For each of the 62 concepts, we zeroed out its activation across all test samples and measured the resulting change in macro F1-score. Because zeroing a concept constitutes an out-of-distribution perturbation, we interpret these analyses as functional probes rather than causal tests. A substantial F1 drop upon ablation indicates that the concept encodes information functionally relevant to classification. We report the mean F1 change across all 5-folds.

### Evaluation metrics

We evaluated models using macro-averaged F1-score (primary metric), accuracy, macro-averaged recall as a sensitivity-oriented clinical metric, specificity, and area under the receiver operating characteristic curve (AUC). Per-class F1-scores were computed for SSL, conventional, and malignant categories. All metrics are reported as mean ± standard deviation across 5-folds. For the primary comparison between the E2E-CBM and black-box baseline, we additionally computed 95% confidence intervals using the *t* distribution over the 5-folds and performed paired fold-level comparisons using a paired *t*-test and Wilcoxon signed-rank test. Confusion-matrix-derived clinical metrics are summarized for the primary matched comparison.

## Results

### Main classification results

Table 1 summarizes the main classification results. Under identical training conditions (batch size 8, 50 epochs), the proposed E2E-CBM with BiomedCLIP alignment achieved a macro F1-score of 95.20% (±1.89%; 95% CI 92.58–97.82%), effectively matching the black-box EfficientNet-B3 baseline of 94.97% (±2.52%; 95% CI 91.48–98.47%). Given the modest absolute difference and overlapping fold variability, we interpret this result as evidence that interpretability can be introduced without measurable performance degradation rather than as definitive superiority over the baseline. The mean paired F1 difference was +0.23 percentage points (95% CI −2.62 to 3.08 percentage points), and paired fold-level testing did not show a statistically significant difference between the two models (paired *t*-test, *p* = 0.835; Wilcoxon signed-rank test, *p* = 1.000). Notably, the CBM exhibited lower variance across folds (SD: 1.89% vs. 2.52%), which may indicate more stable generalization but should be interpreted cautiously given the small number of folds. The baseline achieved a marginally higher AUC (99.13% vs. 98.64%), likely reflecting the unconstrained feature space available to the black-box model. Extending training to 80 epochs further improved CBM performance to 95.49% (±1.72%). This 80-epoch configuration is reported as a sensitivity analysis rather than the primary matched comparison because the black-box baseline was trained for 50 epochs. The CBM without concept alignment (λ = 0) achieved only 92.90% (±2.04%), suggesting that concept alignment is important for optimal performance within the CBM family.

**Table 1:** Main classification results.

Method	λ	F1 (%)	Acc (%)	Sens. (%)	AUC (%)	Interp.
Baseline (black-box)	–	94.97 ± 2.52	94.88 ± 2.53	95.14 ± 2.44	**99.13**±**0.42**	No
**E2E-CBM** **+** **BiomedCLIP**	**1.0**	**95.20** ± **1.89**	**95.08** ± **1.96**	**95.21** ± **1.97**	98.64 ± 0.74	**Yes**
E2E-CBM + BiomedCLIP^†^	1.0	95.49 ± 1.72	95.28 ± 1.91	95.58 ± 1.76	99.02 ± 0.79	Yes
E2E-CBM + OpenAI CLIP^‡^	0.1	93.78 ± 1.99	93.70 ± 2.13	93.87 ± 1.96	98.96 ± 0.58	Yes
E2E-CBM (no alignment)^‡^	0.0	92.90 ± 2.04	92.72 ± 2.21	92.97 ± 1.95	98.66 ± 0.66	Yes

All models use EfficientNet-B3. The primary matched-training CBM configuration is shown in bold. Sens. denotes macro-averaged recall. Confusion-matrix-derived class-wise specificity values for the primary matched comparison are summarized in the text. †: trained for 80 epochs. ‡: trained with batch size 16.

Per-class analysis of the primary CBM configuration revealed strong performance across all categories: SSL F1 of 95.11% (±2.38%), conventional F1 of 92.87% (±2.97%), and malignant F1 of 97.63% (±1.34%). The primary CBM achieved a macro-averaged recall of 95.21% (±1.97%), compared with 95.14% (±2.44%) for the black-box baseline. Further analysis of the pooled predictions showed strong class-wise specificity for both models. The black-box baseline achieved specificity values of 98.73% for SSL, 95.56% for conventional polyps, and 98.07% for malignant lesions. The proposed E2E-CBM showed comparable or slightly improved specificity values of 96.84% for SSL, 96.75% for conventional polyps, and 98.90% for malignant lesions. Detailed pooled confusion matrices are provided in Supplementary material.

### Effect of concept alignment strength

**Figure 2:**
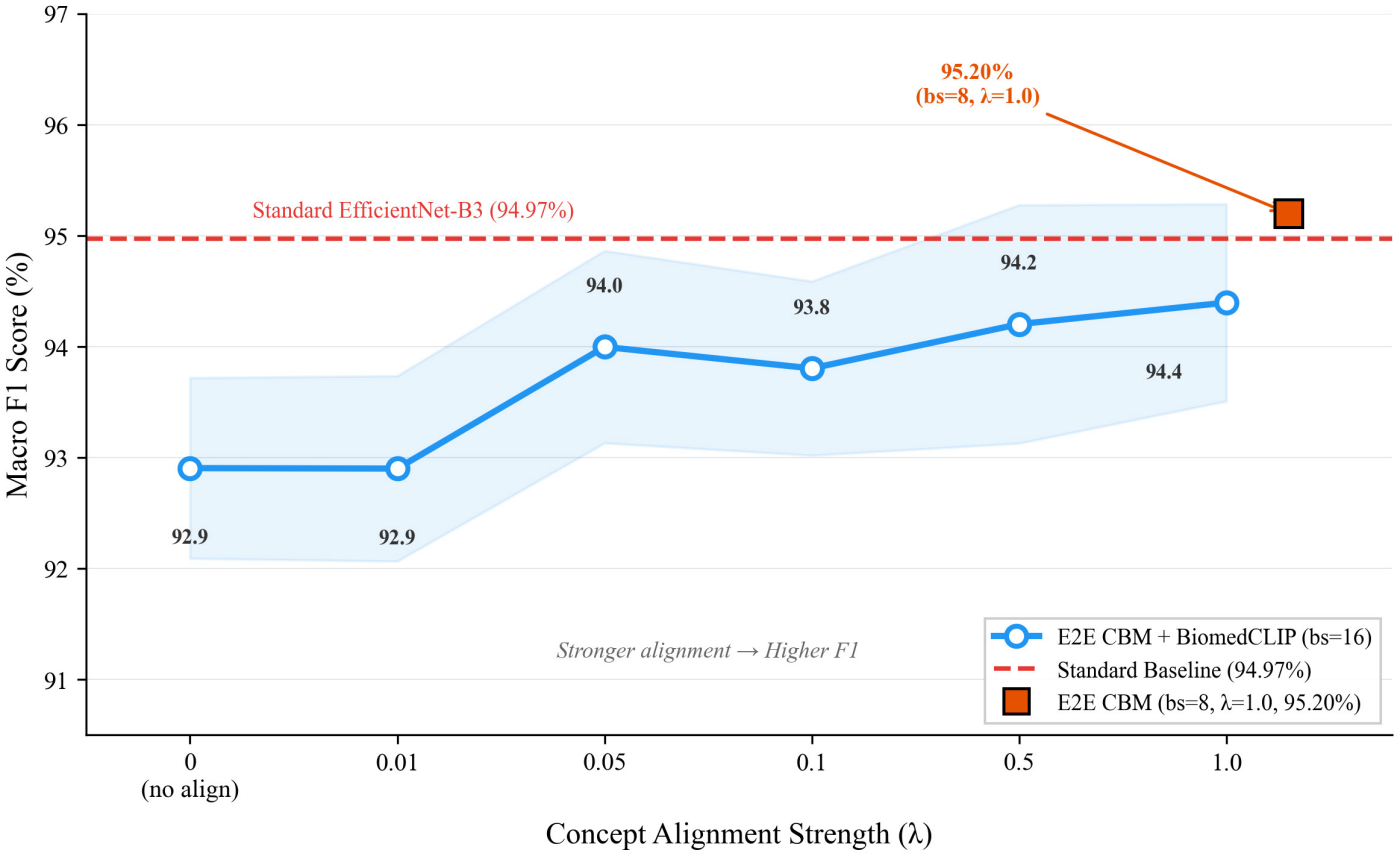
Effect of concept alignment strength (λ) on macro F1-score. Performance shows an overall upward trend from λ = 0 (no alignment, 92.90%) to λ = 1.0 (94.39%). The orange square indicates the best configuration (batch size 8, λ = 1.0, F1 = 95.20%). Shaded bands represent ±0.4 standard deviation across 5-folds.

We investigated the effect of the alignment strength hyperparameter by sweeping from 0 to 1.0 (Table 2 and Figure 2). Performance showed an overall upward trend with increasing, with F1 rising from 92.90% (±2.04%) to 94.39% (±2.22%), a gain of 1.49 percentage points. Although the trend was not strictly monotonic at every adjacent setting, the overall pattern suggests that concept alignment may act as a regularization-like constraint: stronger alignment with clinically meaningful pseudo concept targets may reduce overfitting and improve generalization. Per-class analysis revealed that the largest improvements occurred for SSL (92.39% to 94.43%) and conventional polyps (90.35% to 92.14%), whereas malignant classification remained consistently high.

**Table 2:** Effect of alignment strength λ on classification performance.

λ	F1 (%)	Acc (%)	AUC (%)	SSL F1	Conv F1	Malig F1
0.00	92.90 ± 2.04	92.72 ± 2.21	98.66 ± 0.66	92.39	90.35	95.97
0.01	92.90 ± 2.09	92.71 ± 2.13	98.68 ± 0.84	92.69	89.72	96.29
0.05	93.99 ± 2.17	93.89 ± 2.29	98.78 ± 0.75	94.45	91.57	95.97
0.10	93.80 ± 1.96	93.70 ± 2.13	98.81 ± 0.77	94.13	91.34	95.94
0.50	94.20 ± 2.69	94.09 ± 2.80	98.83 ± 0.65	94.47	91.53	96.61
1.00	94.39 ± 2.22	94.29 ± 2.29	98.82 ± 0.61	94.43	92.14	96.62

All experiments use batch size 16, 50 epochs, ImageNet pre-trained EfficientNet-B3 with BiomedCLIP alignment.

### Ablation study

**Figure 3:**
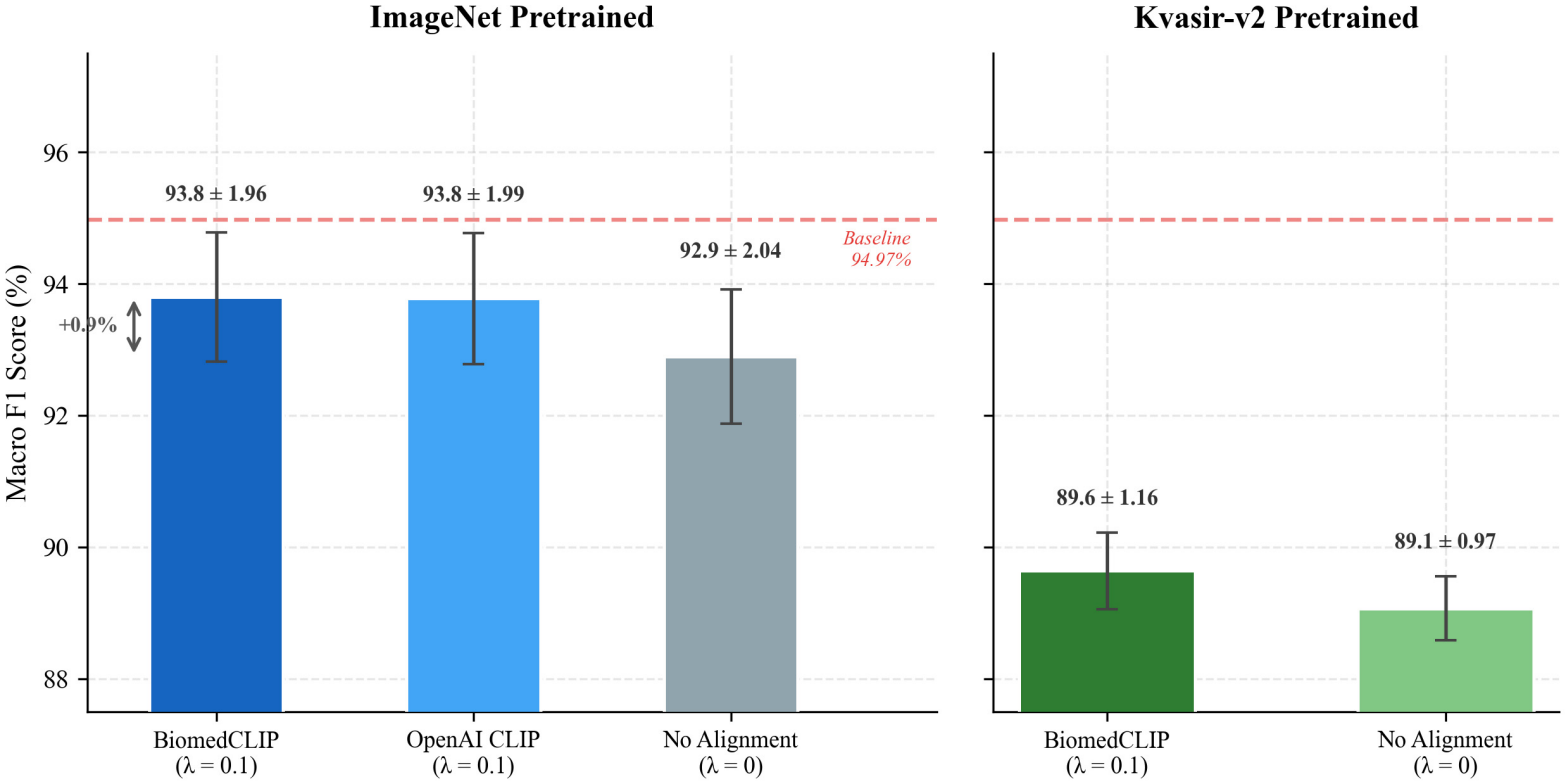
Ablation study examining the effects of backbone architecture (EfficientNet-B3 vs. ResNet-50), pre-training strategy, and concept alignment type on classification performance. Left panel: ImageNet-pre-trained models. Right panel: Kvasir-v2 domain-pre-trained models. The dashed red line indicates the standard EfficientNet-B3 baseline (F1 = 94.97%). Concept alignment consistently improves performance across both backbones.

Table 3 and Figure 3 present an ablation study examining the effects of backbone architecture, pre-training strategy, and vision-language model choice. Four key observations emerged from this analysis. First, EfficientNet-B3 substantially outperformed ResNet-50 (best F1: 95.20% vs. 90.09%), indicating the importance of backbone capacity for this task. Second, concept alignment consistently improved performance across both backbones: +0.90 percentage points for EfficientNet-B3 (92.90% → 93.80%) and +0.32 percentage points for ResNet-50 (89.77% → 90.09%), supporting the potential generalizability of the proposed approach. Third, ImageNet pre-training substantially outperformed domain-specific Kvasir-v2 pretraining (F1: 93.80% vs. 89.64%), likely because general-purpose features learned from ImageNet are more transferable given the limited size of our dataset. Fourth, BiomedCLIP and OpenAI CLIP produced comparable alignment quality (93.80% vs. 93.78%), suggesting robustness to the choice of vision-language backbone.

**Table 3:** Ablation study: backbone architecture, pre-training strategy, and CLIP model choice.

Backbone	Pre-train	CLIP model	λ	F1 (%)	Acc (%)	AUC (%)
EfficientNet-B3						
	ImageNet	BiomedCLIP	0.1	93.80 ± 1.96	93.70 ± 2.13	98.81 ± 0.77
	ImageNet	OpenAI CLIP	0.1	93.78 ± 1.99	93.70 ± 2.13	98.96 ± 0.58
	ImageNet	None	0.0	92.90 ± 2.04	92.72 ± 2.21	98.66 ± 0.66
	Kvasir-v2	BiomedCLIP	0.1	89.64 ± 1.16	89.57 ± 1.18	97.55 ± 0.79
	Kvasir-v2	None	0.0	89.07 ± 0.97	88.97 ± 1.16	97.51 ± 0.62
ResNet-50						
	ImageNet	BiomedCLIP	1.0	90.09 ± 1.59	89.77 ± 1.58	97.60 ± 0.63
	ImageNet	BiomedCLIP	0.1	89.80 ± 3.42	89.57 ± 3.42	97.34 ± 0.63
	ImageNet	None	0.0	89.77 ± 2.49	89.57 ± 2.53	97.49 ± 0.97

Batch size 16, 50 epochs.

### Concept intervention analysis

**Figure 4:**
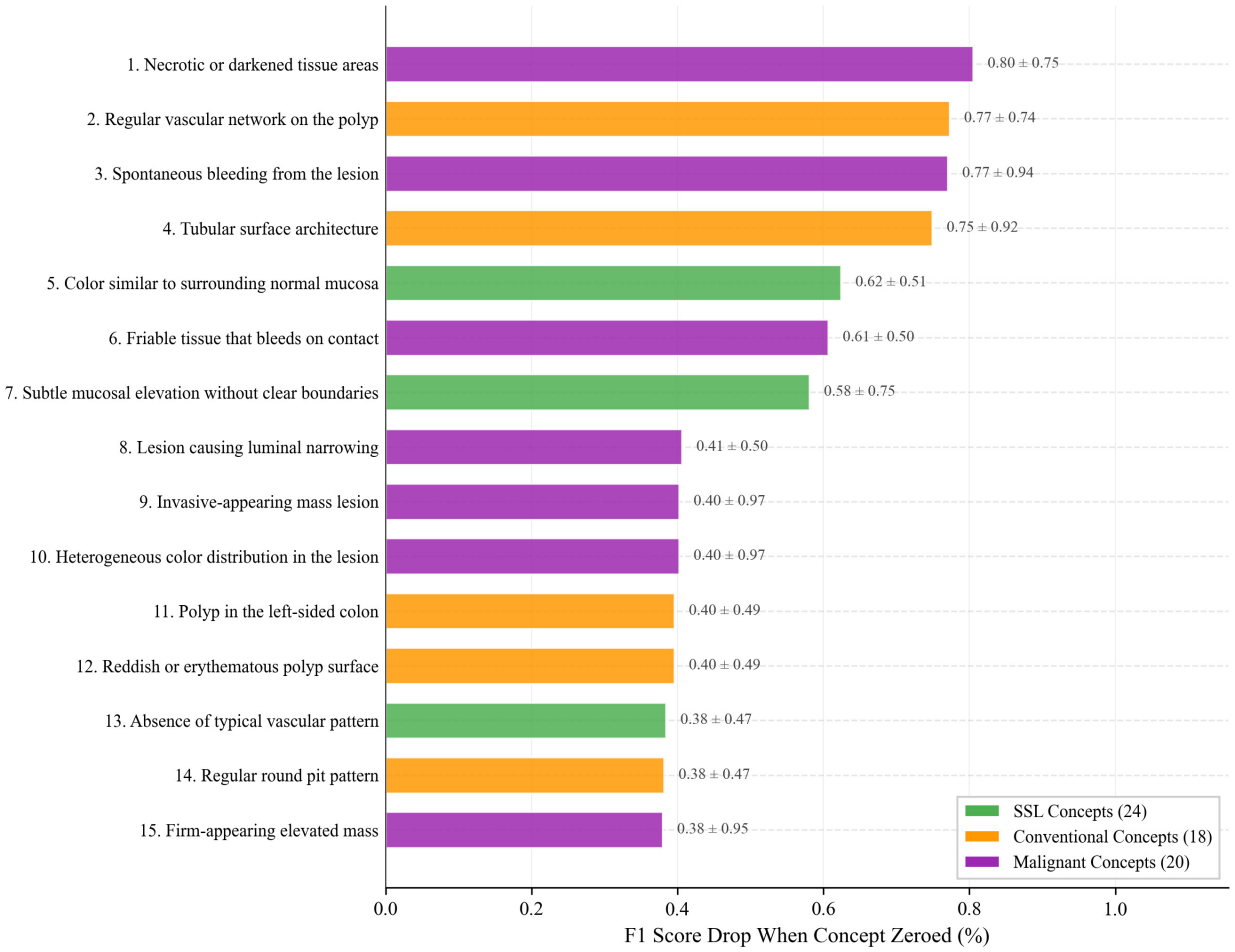
Concept intervention analysis showing the top 15 most influential concepts ranked by F1 score drop when each concept is individually zeroed out. The intervention was performed on the batch-size-16, λ = 0.1 configuration (baseline F1 = 93.79%). Concepts related to malignant features (necrotic tissue, spontaneous bleeding) and vascular patterns (regular vascular network, tubular architecture) exhibit the largest performance drops. Values shown as mean ± standard deviation across 5-folds.

Figure 4 and Table 4 present the results of concept intervention experiments conducted on the batch-size-16, λ = 0.1 configuration (baseline F1: 93.79%). We treat these interventions as functional probes rather than causal tests because zeroing a concept constitutes an out-of-distribution perturbation. Zeroing out individual concepts produced changes in F1 ranging from −0.80% to +0.40%, with no single concept exceeding a 1 percentage point impact. This distributed encoding pattern indicates that the model relies on multiple concepts simultaneously rather than on any single discriminative feature, mirroring the multi-feature reasoning employed by endoscopists in clinical practice.

**Table 4:** Top-10 most influential concepts identified by intervention analysis.

Rank	Concept	F1 drop (%)	Class
1	Necrotic or darkened tissue areas	−0.80 ± 0.75	Malignant
2	Regular vascular network on the polyp	−0.77 ± 0.74	Conventional
3	Spontaneous bleeding from the lesion	−0.77 ± 0.94	Malignant
4	Tubular surface architecture	−0.75 ± 0.92	Conventional
5	Color similar to surrounding normal mucosa	−0.62 ± 0.51	SSL
6	Friable tissue that bleeds on contact	−0.61 ± 0.50	Malignant
7	Subtle mucosal elevation w/o clear boundaries	−0.58 ± 0.75	SSL
8	Lesion causing luminal narrowing	−0.41 ± 0.50	Malignant
9	Invasive-appearing mass lesion	−0.40 ± 0.97	Malignant
10	Heterogeneous color distribution	−0.40 ± 0.97	Malignant

F1 drop indicates the decrease in macro F1-score when the concept is ablated.

**Figure 5:**
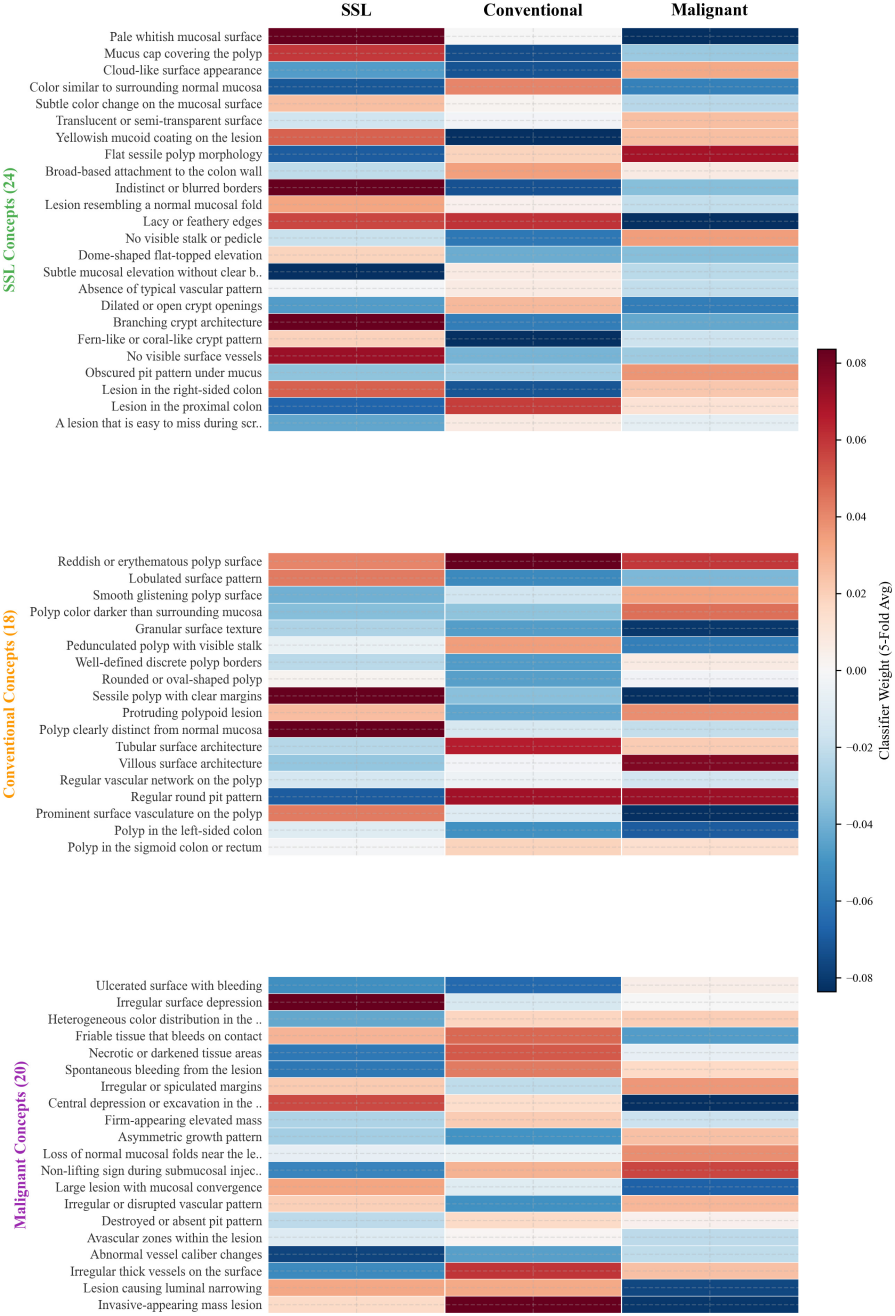
Concept-to-class weight matrix learned by the linear classifier, averaged across 5-folds. Each row represents one of 62 clinical concepts, and each column represents a polyp class (SSL, Conventional, Malignant). Red indicates positive weights (concept supports the class prediction) and blue indicates negative weights (concept opposes the class prediction). The model learns clinically interpretable patterns: SSL concepts (e.g., pale surface, flat morphology) positively contribute to SSL classification, while malignant concepts (e.g., necrotic tissue, irregular vessels) positively contribute to malignant classification.

The most influential concepts corresponded to clinically recognizable features: “necrotic or darkened tissue areas” (−0.80%), “regular vascular network” (−0.77%), “spontaneous bleeding” (−0.77%), and “tubular surface architecture” (−0.75%) ranked highest. Among SSL-specific concepts, “color similar to surrounding normal mucosa” (−0.62%, rank 5) and “subtle mucosal elevation without clear boundaries” (−0.58%, rank 7) were most influential, consistent with the known diagnostic criteria for SSLs. Malignant-associated concepts dominated the top 10, reflecting the visually distinct features characteristic of malignant lesions. The concept-to-class weight matrix (Figure 5) further illustrates how the linear classifier learned clinically plausible associations between concepts and polyp categories.

### Qualitative analysis

**Figure 6:**
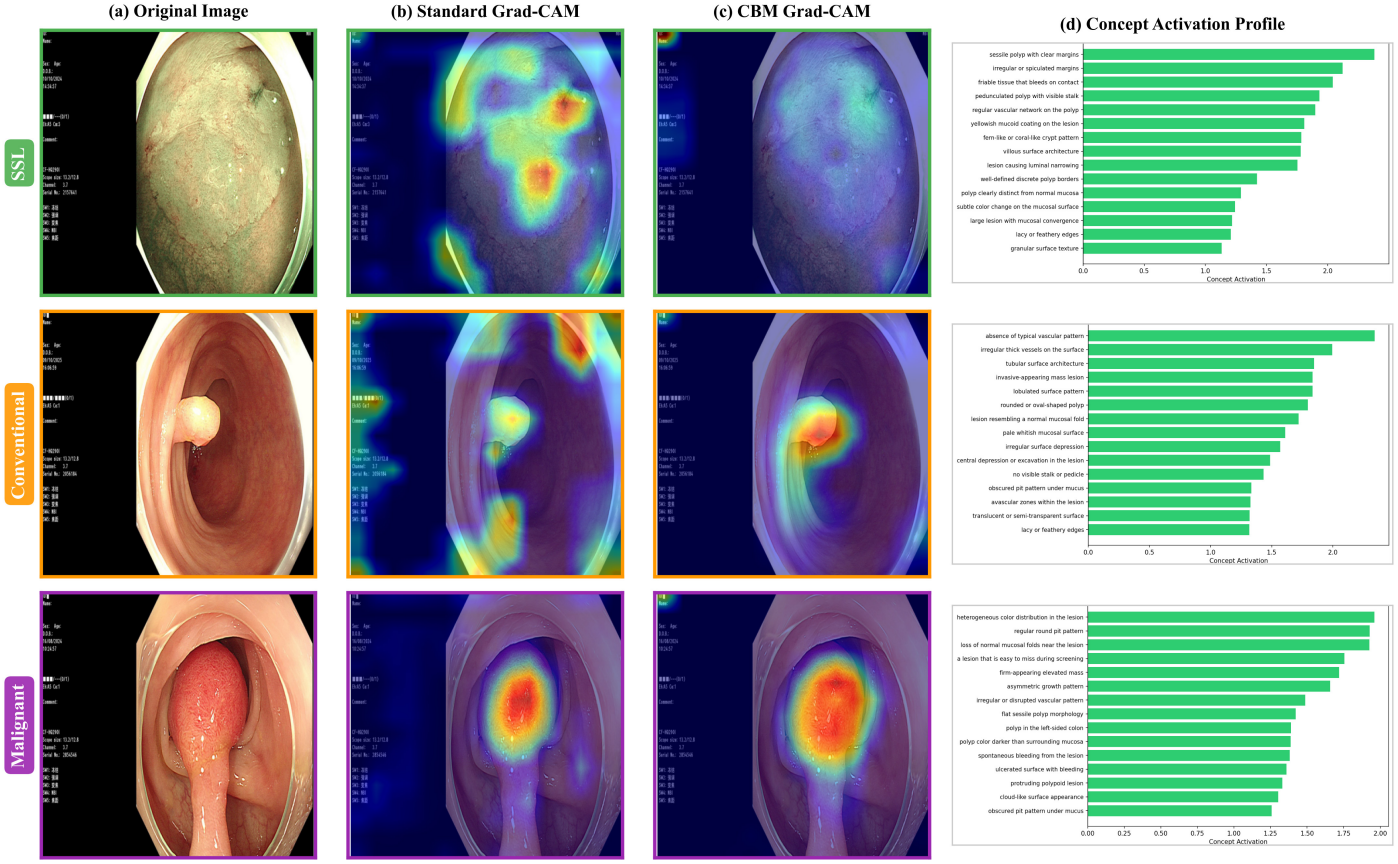
Concept-to-class weight matrix learned by the linear classifier, averaged across 5 folds. The three concept groups are displayed as stacked panels from top to bottom: SSL concepts (24), Conventional concepts (18), and Malignant concepts (20). Within each panel, each row represents one clinical concept and each column represents a polyp class (SSL, Conventional, Malignant). Red indicates positive weights (concept supports the class prediction) and blue indicates negative weights (concept opposes the class prediction). The model learns clinically interpretable patterns: SSL concepts (e.g., pale surface, flat morphology) positively contribute to SSL classification, while malignant concepts (e.g., necrotic tissue, irregular vessels) positively contribute to malignant classification.

Figure 6 compares Grad-CAM visualizations between the standard black-box model and the proposed CBM across representative samples from each class. Both models focus attention on the polyp regions, demonstrating comparable spatial localization. The CBM provides an additional layer of explanation through concept activation profiles, which indicate *what* model-aligned clinical descriptors are activated in each image. For instance, SSL cases showed high activation for “pale whitish surface,” “indistinct borders,” and “mucus cap”—features corresponding to established SSL diagnostic criteria. These qualitative results should be interpreted as illustrative rather than as clinical validation of the explanations. This concept-level transparency complements saliency maps by linking attention to clinically meaningful descriptors.

## Discussion

### Interpretability as regularization

Our central finding is that concept alignment does not impose an obvious performance penalty and may act as a regularization-like constraint within the CBM framework. The overall upward trend in F1-score with increasing (Table 2) suggests that aligning intermediate representations with clinically meaningful concepts may help constrain representation learning. By constraining the 1,536-dimensional feature space to pass through a 62-dimensional concept bottleneck grounded in domain knowledge, the model may be less prone to learning spurious correlations in the limited training set. This interpretation is conceptually related to information bottleneck theory (Tishby and Zaslavsky, 2015), in which compressing representations to retain only task-relevant information can improve generalization. However, our experiments do not directly prove the mechanism of regularization, and the reduced variance observed in the CBM should be considered suggestive rather than definitive.

### Clinical relevance of learned concepts

The concept intervention results (Table 4) provide evidence that the learned concept representations are clinically plausible. The most influential SSL concepts—“color similar to surrounding normal mucosa” and “subtle mucosal elevation without clear boundaries”—correspond to the very features that make SSLs clinically challenging: their mimicry of normal mucosa and subtle elevation (IJspeert et al., 2016). Similarly, the dominance of bleeding and vascular features among malignant concepts aligns with the JNET classification's emphasis on vascular patterns for malignancy assessment (Sano et al., 2016). The distributed encoding pattern, in which no single concept dominates, mirrors clinical practice, where endoscopists integrate multiple visual features for diagnosis. Nevertheless, because the concept targets were generated by BiomedCLIP rather than by expert annotation, these concept activations should be interpreted as model-derived explanatory signals rather than validated clinical labels.

This concept-level interpretability offers advantages over gradient-based methods such as Grad-CAM (Selvaraju et al., 2017), which indicate spatial attention but cannot specify which morphological or vascular features informed the decision. With our CBM, clinicians can inspect the activated concepts for each prediction and assess whether the model's reasoning is plausibly aligned with clinical guidelines—a crucial step toward future trust in AI-assisted colonoscopy (Topol, 2019).

### Limitations and future work

This study has several limitations. The dataset included 508 images from a single center, and cross-validation was performed at the image level rather than the patient or lesion level because patient and lesion identifiers were not available for the present analysis. Multi-center validation with patient- or lesion-level splits is therefore needed to assess generalizability and reduce the risk of correlated-sample bias. The primary CBM and black-box baseline showed no statistically significant F1 difference in paired testing, so our claim is performance preservation with interpretability rather than superiority. In addition, concept alignment targets were generated by BiomedCLIP rather than expert annotations, which may introduce noise, domain bias, or semantic mismatch; future work should compare VLM-derived concepts with gastroenterologist-annotated concept labels. Future prospective evaluations should preserve full per-sample prediction outputs directly so that specificity, confusion matrices, and false-positive patterns can be audited in greater detail. The current three-class setting did not include non-polyp or out-of-distribution images; therefore, false-positive behavior against normal mucosa or unrelated endoscopic findings could not be fully assessed. Future studies should introduce a fourth non-polyp class and additional out-of-distribution samples to directly evaluate false-positive behavior in real-world colonoscopy screening. Real-world factors such as lighting variation, shadows, pigmentation differences, mucus, and motion blur may also affect SSL interpretation and should be evaluated in external validation cohorts. Future studies should further refine the concept vocabulary through expert consensus, test additional architectures, and prospectively assess whether concept-based explanations improve clinical decision-making.

## Conclusions

We presented the first end-to-end CBM for colonoscopic polyp classification, demonstrating that interpretability can be incorporated without sacrificing performance. By aligning model representations with 62 clinically grounded concepts via BiomedCLIP, our framework achieves a macro F1-score of 95.20%, comparable to the black-box baseline while providing transparent, concept-level explanations. The overall improvement trend observed with increasing alignment strength suggests that concept alignment may act as a regularization-like constraint within the CBM framework. Concept intervention analyses suggest that the learned concepts encode functionally relevant information for classification in a distributed manner consistent with clinical reasoning. These findings support the development of more interpretable AI systems for colonoscopy.

## Data Availability

The original contributions presented in the study are included in the article/Supplementary material, further inquiries can be directed to the corresponding author.
