# Supplementary material for: Interpretable polyp classification via end-to-end Concept Bottleneck Models with vision-language concept alignment
Source: Front Artif Intell. 2026 Jul 10;9:1853584. doi: 10.3389/frai.2026.1853584 (PMC13397497; doi:10.3389/frai.2026.1853584)
Supplement: Supplementary file 1 [file Data_Sheet_1.pdf]

Supplementary Material: Pooled Confusion Matrices and Class-wise Clinical Metrics

Manuscript ID: 1853584

This supplementary material provides the pooled confusion matrices and class-wise sensitivity/specificity values for the primary matched comparison reported in the revised manuscript. Rows represent true labels and columns represent predicted labels, ordered as SSL, conventional polyp, and malignant lesion.

Pooled Confusion Matrices

Model	Pooled confusion matrix, rows=true and columns=predicted (SSL/ Conventional/Malignant)
Black-box EfficientNet-B3	177, 12, 3; 4, 162, 4; 0, 3, 143
E2E-CBM + BiomedCLIP	183, 8, 1; 10, 157, 3; 0, 3, 143

Class-wise Sensitivity and Specificity

Model	SSL sensitivity/ specificity (%)	Conventional sensitivity/ specificity (%)	Malignant sensitivity/ specificity (%)
Black-box EfficientNet-B3	92.19 / 98.73	95.29 / 95.56	97.95 / 98.07
E2E-CBM + BiomedCLIP	95.31 / 96.84	92.35 / 96.75	97.95 / 98.90

These values correspond to the primary matched five-fold validation comparison between the black-box EfficientNet-B3 baseline and the proposed E2E-CBM with BiomedCLIP concept alignment.
